# Artificial Lipid Membranes: Past, Present, and Future

**DOI:** 10.3390/membranes7030038

**Published:** 2017-07-26

**Authors:** Christina G. Siontorou, Georgia-Paraskevi Nikoleli, Dimitrios P. Nikolelis, Stefanos K. Karapetis

**Affiliations:** 1 Laboratory of Simulation of Industrial Processes, Department of Industrial Management and Technology, School of Maritime and Industry, University of Piraeus, 18534 Piraeus, Greece; csiontor@unipi.gr; 2 Laboratory of Inorganic & Analytical Chemistry, School of Chemical Engineering, Department of Chemical Sciences, National Technical University of Athens, 15780 Athens, Greece; tzwrtzia85@hotmail.com (G.-P.N.); stevekara@chem.uoa.gr (S.K.K.); 3 Laboratory of Environmental Chemistry, Department of Chemistry, University of Athens, 15771 Athens, Greece

**Keywords:** artificial lipid membranes, liposomes, Langmuir-Blodgett films, tethered membranes, self-assembly, nanoelectrodes, biosensors, drug delivery, ion channel monitoring, artificial cells

## Abstract

The multifaceted role of biological membranes prompted early the development of artificial lipid-based models with a primary view of reconstituting the natural functions in vitro so as to study and exploit chemoreception for sensor engineering. Over the years, a fair amount of knowledge on the artificial lipid membranes, as both, suspended or supported lipid films and liposomes, has been disseminated and has helped to diversify and expand initial scopes. Artificial lipid membranes can be constructed by several methods, stabilized by various means, functionalized in a variety of ways, experimented upon intensively, and broadly utilized in sensor development, drug testing, drug discovery or as molecular tools and research probes for elucidating the mechanics and the mechanisms of biological membranes. This paper reviews the state-of-the-art, discusses the diversity of applications, and presents future perspectives. The newly-introduced field of artificial cells further broadens the applicability of artificial membranes in studying the evolution of life.

## 1. Introduction

Lipid membranes have a key role to play in most physiological processes: cell protection, cell-to-cell communication, between-cell and within-cell control of micro-environments, and metabolism. Although membranes have been intensively studied and exploited over the last 40 years, the full-scale and depth of their structural organization and the interrelation between all components at the molecular level remain largely elusive. For instance, the two leaflets of the plasma membrane do not have similar phospholipid composition: aminophospholipids are mostly found at the inner (cytoplasmic) leaflet whereas cholinephospholipids preferentially occupy the outer (exoplasmic) leaflet [1]. This asymmetry serves certain functions, such as the formation of a procoagulant interface upon cell disruption to trigger recognition events, the regulation of membrane budding, or the structural stability [2]; yet, it is not clear how this asymmetry initially arises.

As instrumentation evolves towards more sophisticated tools for research, certain aspects on membranes are revisited and theories are heavily tested. The lipid rafts hypothesis, i.e., the existence of ordered liquid crystalline lamellar phase microdomains among less-ordered lipid areas, is a case in point. Using detergent extraction of membrane lipids, these microdomains resist solubilization in non-ionic media whereas the less ordered surroundings do not [3]. This test helped to produce a conceptual architecture that explains satisfactorily the lateral protein distribution, the localization of various constituents in small compartments to allow their interaction, and the dynamics of the lipid backbone in terms of mobility. It was further used as the theoretical basis for explaining membrane fusion, enzyme catalysis, molecular recognition, and cellular adhesion. Later it became evident that detergent testing is not reliable because non-ionic detergents per se might affect the organization of lipids to induce the formation of such microdomains [4]. The existence of submicron lateral heterogeneities have been, however, confirmed by thermodynamic phase diagrams [4], nuclear magnetic resonance [5], confocal microscopy and fluorescence correlation spectroscopy [6], and photon fluorescence microscopy [7],but whether their existence supports biological processes or the biological processes induce their formation is not yet fully elucidated [8]. Recent evidence, however, provided by spherically supported bilayer lipid membranes, strongly suggests that the dynamic organization of the lipid membrane is indeed a function of lipid microdomains [9].

In effect, membranes have proven to be complex and strictly regulated systems with functions depending upon changes of a meta-stable and highly dynamic lipid bilayer core framework [10,11,12]. The dimensions of the bilayer, a few nanometers in width and tenths of micrometers in length (a 2D nanomaterial according to all available definitions), inspired and intrigued researchers and engineers, especially as regards the physics behind its formation and properties: self-assembled, free-standing, thermotropic, and self-repairing. Self-assembly proved extremely valuable, as it allowed the formation of model membranes using relatively simple laboratory set-ups. Artificial lipid membranes could be constructed and experimented upon since the early 1960s (for a recent review see [13]). These models served effectively and in parallel two scopes: elucidation of physiological mechanisms and niche applicability in therapeutics and metrology. There exists the most prominent example of a continuously back and forth transition between science and technology, with rates escalating at the advent of the nano era.

This paper discusses critical parameters of membrane mimicking platforms, putting emphasis on their diverse applicability as both, tools for research and platforms for detection, drug discovery, and drug testing. Current design and construction approaches, state-of-the art modularization, and fictionalization are also presented, along with insights on the future perspectives of cell mimicking.

## 2. Lipid Membrane Platforms: Definitions, Configurations, and Stability

Given that membrane function is the product of the way it is formed and sustained in thermodynamic terms, the definition of lipid membranes has to include at least three aspects: composition, molecular dynamics, and phase behavior. A membrane is a two-layer organization of amphipathic molecules, most commonly of lipids (glycerophospholipids, sphingolipids, sterols) although a variety of lipid-like materials has been also proposed. Jin et al. [14] constructed membrane mimics from self-assembling two dimensional nanoscale lipid-like peptoids. The resultant film, having a thickness of 3.5–5.6 nm and including hydrophobic areas surrounded by hydrophilic surfaces, changes its thickness in response to external stimuli and self-repairs minor defects. The incorporation of ion channels and proteins is the next research stage in producing membrane-mimic analogues. Terrettaz et al. [15] published a comprehensive report on the incorporation of ion channel proteins in lipid vesicles for sensing applications.

**Figure 1:**
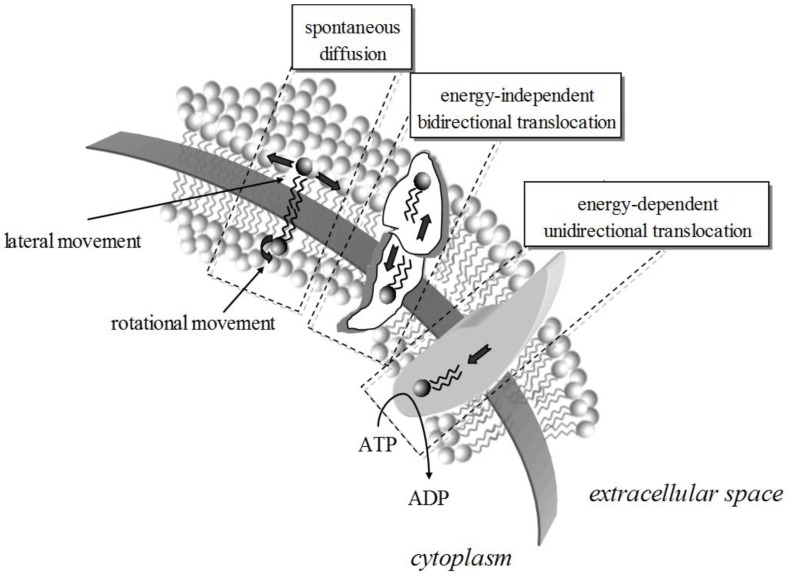
The movement of lipids within a bilayer takes many forms, energy-driven or spontaneous. The rates for rotational and lateral movement depend on the biophysics of the membrane and the lipids. Lipid translocation from one leaflet to the other can be protein-mediated and energy independent (e.g., via scramblases) or energy-dependent (e.g., via translocases).

Lipid dynamics are defined by the lateral and rotational diffusion coefficients determining the position and orientation shifting of lipids at any given leaflet [8] (Figure 1). Conformational changes may also occur leading to or resulting from lipid shifting. This kind of mobility is fast, with a pico- to milli-seconds duration. Molecular dynamics are demonstrated during the exchange of lipids between the two leaflets (flip-flop); as the polar group has to pass through the hydrophobic area to end up at the other side, this transversal movement is slow and complex. Commonly seen with cholesterol, this motion is thermodynamically unfavorable for lipids with higher than one hydroxyl group polar head [16].

Phase shifting is critical to the membrane system. Besides the ordered (gel) and disordered (liquid) phases, less-ordered (gel-to-liquid) phases may coexist [17,18]. The transition from one phase to the other, i.e., the changes in molecular packing and fluidity, may or may not lead to interdigitation depending on a threshold concentration of the inducer [19]. The interdigitated gel phase is currently applied in the formation of unilamellar vesicles, proved to be successful membrane models with many practical applications [20]. Phase shifting can be induced in model lipid bilayers by surface aggregation phenomena driven by immunological reactions [21] or DNA adsorption [22]. Phase segregation has been also linked to protein sorting and signaling, lipid self-assembly to bilayer, pore formation, and curvature [23].

**Figure 2:**
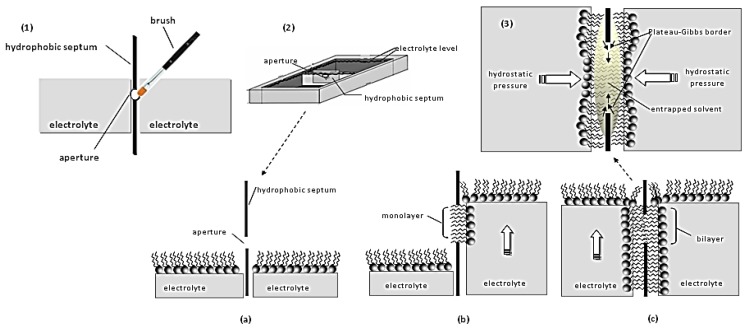
Schematic of conventional approaches for preparing freely-suspended bilayer lipid membranes: (**1**) Painting technique: A droplet of lipid solution is painted into a small aperture in a hydrocarbon or Teflon partition; the thinning of the lipids into a bilayer occurs spontaneously (black lipid film). (**2**) Folding or monolayer opposition technique: (**a**) A hydrophobic septum, punched to produce an aperture of a few millimeters, separates two electrolyte compartments. (**b**) The electrolyte is removed from both compartments; lipids are added and accumulate at the surface of the aqueous phase. When the electrolyte level is raised in one compartment, one lipid monolayer is forced to develop around the aperture. (**c**) Replenishing the other compartment with electrolyte, attaches the second monolayer (like zipping); during zipping the solvent entrapped into the hydrophobic area is squeezed by the hydrostatic pressure applied on both sides of the bilayer towards the rim of the aperture (Plateau-Gibbs border). (**3**) Finally, an equilibrium is reached between membrane thinning (due to hydrostatic pressure) and unzipping (due to the solvent that tries to relocate at the middle of the bilayer). As evident, the slightest vibration disturbs this equilibrium and the bilayer collapses.

**Figure 3:**
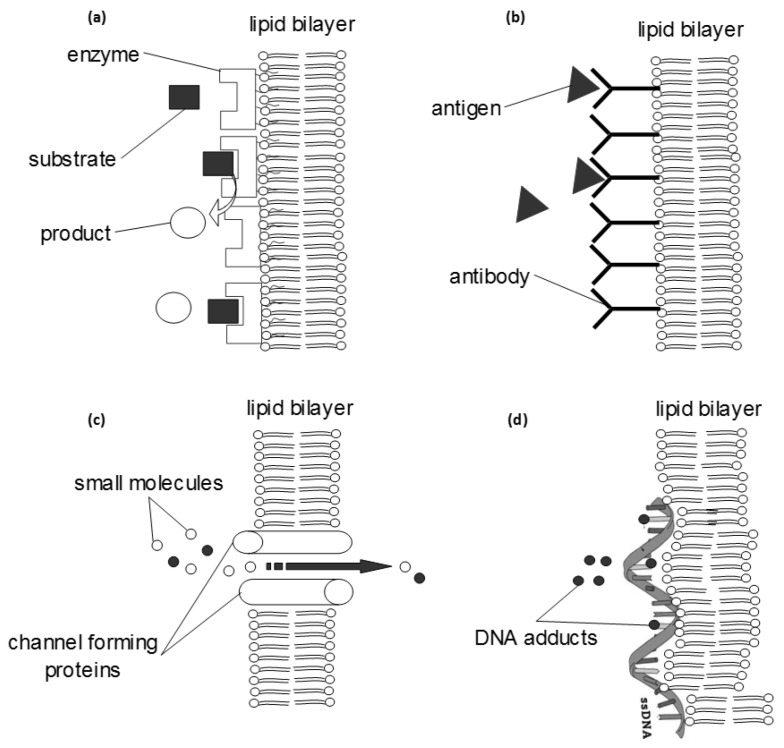
Some basic biochemical systems that can be reconstituted within an artificial lipid bilayer and further engineered into a diagnostic system (biosensor). The transduction of the biochemical information into a detector signal can be achieved with electrochemical, optical, piezoelectric or magnetic sensors. (**a**) bioaffinity interfaces can be constructed using enzymes or receptors, adsorbed on the membrane surface; the whole system can be optimized electrochemically (using redox amplifiers) or optically (using fluorescent tags). (**b**) The monitoring of immunochemical reactions follows similar methodology and further allows for the development of more advanced and rapid signal propagation systems (e.g., using enzymes, tagging or radiochemistry). (**c**) Many channel-forming proteins have been reconstituted within a bilayer; small molecules can flow through the channel, but in most cases some selectivity rules apply that make possible the development of a detection system (e.g., gramicidin channels transport potassium ions faster than sodium while valinomycin channels transport only potassium ions). (**d**) Single- or double-stranded DNA can adsorb on the membrane surface to investigate the effects of various adducts or to detect mutagens.

It is widely acknowledged that the functionality of any artificial membrane is built-in during its formation, i.e., the thermodynamics that hold it in place also govern the way it interacts with its surrounding. Self-assembly is the simplest way to produce a membrane as long as a suitable drive is provided. Spreading lipids (in organic solvent) across an aperture that separates two aqueous phases, forces them to organize themselves into two leaflets, orienting their hydrophilic heads in each side towards the solution in order to keep the hydrophobic tails enclosed [24,25,26,27,28] (Figure 2). The formation is sustained by the interplay between two forces: the hydrodynamic force that tries to compress the two leaflets in the horizontal axis and the solvent stored at the edge of the aperture (Plateau-Gibbs border) that tries to separate them at the perpendicular axis. The bilayer controls ion mobility between the two aqueous phases, i.e., the current flux (transmembrane current), while building up dipolar potential perpendicular to the membrane plane [29]. The polar region attracts ions that accumulate at the membrane surface giving rise to a surface charge. Any electrochemical change at the interface between the membrane surface and the solution (at Debye length) alters the dipolar potential, the surface charge or the packing of lipids (or any combination of the above), resulting in the formation of (temporary or permanent) pores or channels, i.e., small defects through the hydrophobic continuum, that allow the passage of ions to the other side of the membrane [13]; electrical potential, capacitance, current, and elasticity changes can be readily monitored with two- or three-electrode systems. As biochemical interactions occurring at the membrane surface produce such electrochemical changes, any affinity system (substrate-enzyme, antigen-antibody, ion channel activity, substrate-receptor, DNA, etc.) can be reconstituted within these lipid platforms to yield sensor set-ups (Figure 3).

These freely-suspended platforms proved to be successful simulators of membrane physics but their inherent fragility prevented any further attempt in engineering robust detection systems [30]. Yet, this fragility arises from the ‘fluidity’ of the membrane, a dynamic property that accounts for the major part of its functionality. Using model membranes, two instability statuses could be observed: rupture and buckling [31]. The former can be seen during pore formation and fragmentation, as a result of local perturbations of lipid organization [32]; the latter gives rise to membrane bending or folding due to leaflet asymmetry or membrane tension modifications [33]. Protein movement actually relies on the ability of the membrane to become unstable, i.e., to increase its free energy with respect to certain thermodynamic parameters, while keeping other parameters stable so that the overall rate of change remains low. It can be therefore suggested that membrane instability is very difficult to define in thermodynamic terms and unless changes are dramatic (e.g., at cell lysis) one cannot discriminate between an unstable, non-functional membrane and a stable, functional membrane that is simply changing its state.

Constructing lipid membranes on various supports provides better mechanical stability to allow for more vigorous experimentation. Self-assembly remains the most preferred method for construction, although the exact orientation of embodied features cannot be controlled or accurately reproduced. Various materials can serve as supports (gels, mica, ceramics, metals or silicon).

**Figure 4:**
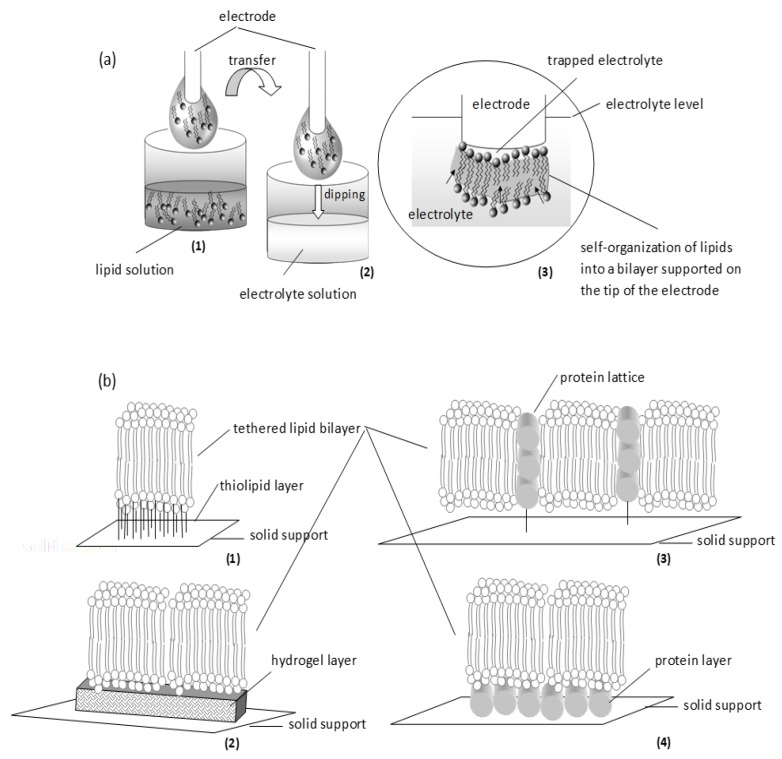
Schematic of two simple approaches in supporting the bilayer: self-assembly (**a**) and tethering (**b**). (a) Self-assembly: (1) A metal wire (freshly cut) is dipped into lipid solution; when withdrawn, a small drop of lipid solution is attached around the tip. (2) The electrode is immersed in electrolyte solution. (3) On dipping into the aqueous phase, lipids gather spontaneously at the tip of the electrode (pushing along electrolyte molecules) to form a monolayer that drives other lipid molecules to cover it at the top; the assembly is finally thinned to a bilayer. (b) Tethering: Thiolipids (1) or hydrogels (2) can be used as the anchoring layer. Alternatively, proteins can be used either as a lattice (3) or as a layer (4).

The dipping method is the simplest way to construct a bilayer platform onto the tip of a metal (Ag, Pt, Ni, etc.) wire: when the wire is dipped into the lipid solution, a small drop of the solution adheres at the wire edge; when the wire is transferred into an electrolyte solution, the lipid drop is forced to self-organize into a bilayer at the wire tip (Figure 4). This is possibly a two-step process [34]: as the wire is immersed into the electrolyte, a monolayer is attached onto the support through colloidal interactions, pushing and trapping a small amount of water between the metal and the polar area of the lipid layer; when the wire is fully immersed dispersed lipid molecules form the second monolayer on top of the first. The resulting membrane is asymmetric in terms of fluidity: the mobility of lipids at the inner leaflet (those in contact with the metal) is restricted, whereas the lipids at the outer leaflet (those in contact with the electrolyte) can move more freely [35]. Vesicle fusion is a simpler variation of this technique: charged unilamellar vesicles are injected onto an oppositely charged solid support [36]; upon contact with the metal, the vesicles rupture and re-organize into an anchored bilayer. Metal-supported platforms are robust enough to allow the development of various probes or interdigitated electrodes utilizing surface-bound proteins and events [13]. Ionic translocation and transmembrane protein localization cannot be reconstituted at these models.

Langmuir-based technology can be used to develop platforms with exact orientation and placement of membrane components in order to reconstitute surface or transmembrane biochemistry. Both vertical (Langmuir-Blodgett, LB) and horizontal (Langmuir-Schaefer, LS) lifting procedure sallowed the transfer of the Langmuir films to a solid substrate, under optimized transfer ratios. Similar to 3D printing, lipid monolayers compressed at air/water interface are transferred and deposited onto metal supports producing stacks of monolayers to suit any design and engineering need, such as pre-specified layer thickness, extended length, hybrid (i.e., of different lipid composition) membranes, multi-layer structures, and a variety of anchoring options to any support [37]. Defects, such as disclinations, gaps, and phase inhomogeneities, are expected to occur especially towards the edges or the center of the film [38]. Charitat et al. [39] proposed a very interesting variation of this technology in an attempt to produce model membranes for biophysical experimentation: using a combination of LB and LS techniques the authors produced a double deposition scheme with one bilayer adsorbed on a solid substrate and a second bilayer ‘floating’ 2–3 nm away. The floating membranes could be readily interrogated with reflectivity, neutron and X-ray scattering techniques at nanometer resolution (for a comprehensive review, see [40]).

Tethering lipid bilayers to solid supports using spacers (e.g., thiolipids, silanelipids, gold, or even DNA oligomers) allows the construction of more complex and efficient assemblies for monitoring transmembrane activity (Figure 4). Since the tethering part should be somehow insulated from water, proteins or ions, the support must be divided into accessible and inaccessible compartments [41]; this architecture restricts lipid and protein movements. Impedance may be used to monitor multiple channel activity but single-channel recordings cannot be made unless the formed membrane has been designed to high electrical resistance [42]. Alternatively, membranes can be stabilized by a protein film used as a tethering layer to support the membrane on the metal surface or as a lattice to host the membranes inside [43]. This architecture is more suitable to study transmembrane protein function at a single unit level.

Assembling membranes on softer supports may allow a degree of suspension for better physical simulations. Using porous supports (alumina or ultrafiltration glass or polycarbonate filters), a network of interconnected membranes can be formed occupying the pores, supported by the non-porous part of the substrate [13]. The resultant increases membrane area and protein-incorporation capacity manifold. Alternatively, hydrogels, especially chitosan, or polymers (e.g., polyethyleneimine, polyacrylamide, and polysaccharides) could serve as cushions for membranes (Figure 4). This approach supports the membranes in a more natural way but restricts surface chemistry and interfacial phenomena by both, physical obstruction of the surface and a limited ionic reservoir [27].

Microfabrication of lipid bilayers follows two trends: the preparation of the films in micro-apertures (e.g., see [44]) and the use of microfluidics for automated membrane formation using lipid droplets (e.g., see [45]). The former requires the machining of micro-apertures on silicon or other hydrophobic sheets; these apertures can be arranged laterally to produce conventional micro-bilayers or vertically for novel architectures [44]. However, apertures less than 40 μm result in bilayers with a high noise background that prohibits further experimentation [13].

**Figure 5:**
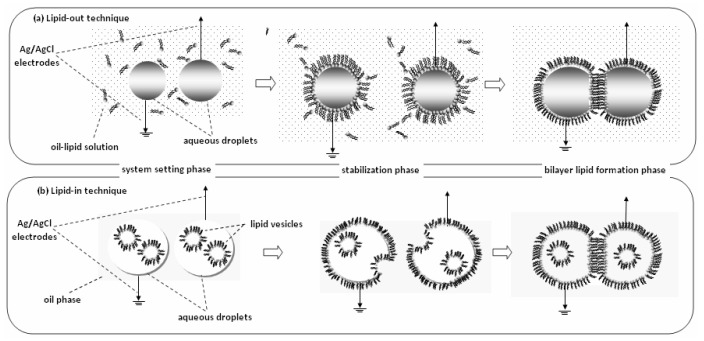
Droplet Interface Bilayers (DIBs) can be formed by two techniques, lipid-out and lipid-in. (**a**) Lipid-out technique: Ag/AgCl electrodes coated with agarose are loaded with aqueous droplets and dipped in oil-lipid solution (system setting phase). A 30-min stabilization phase is necessary for the formation of monolayers at the oil-water interface around the droplets. When the monolayers collide, they form a bilayer at the contact point (bilayer lipid formation phase). (**b**) Lipid-in technique: Similarly, the electrodes are loaded with aqueous droplets that contain vesicles and dipped in oil solution (system setting phase). A 5-min stabilization phase is necessary for the vesicles to fuse with the oil-water interface and form the monolayers, which subsequently brought into contact to form the bilayers.

Droplet interface bilayer (DIB) is a recent technique for automated formation of multiple bilayers in parallel (Figure 5). There are two options for driving the bilayer formation [45]: the deposition of aqueous droplets into a lipid-solvent mixture (lipid-out) or the deposition of lipid vesicles on organic solvent (lipid-in). At any case, lipid monolayer droplets are spontaneously formed around the aqueous surfaces; when they collide, bilayers or higher structures are formed. Both methods include a stabilization phase, the nature of which is still uncertain; it could correspond to the time required for the formation of the monolayers [46] or some kind of gelation process takes place [47]. Lipid-in DIBs undergo a significantly less stabilization period, as droplet mobility through the solvent mixture is faster than the movement of the droplets through the lipid-solvent mixture required in lipid-out DIBs [48]. Further, the lipid-in technique can be used to produce asymmetric bilayers [49], allowing the incorporation of different vesicles in each droplet. These films are mechanically robust and very useful for studying ionic flux through membrane pores seeded within the bilayer [50] or for developing networks to simulate complex biomolecular systems [51].

Nanopatterning methods allow the accurate control of lipid architectures; nanotopography drives bilayer formation in nano-wells, readable with total internal fluorescence microscopy [52]. Still expensive and inconclusive as regards the interpretation of membrane signals, lipid polymerization comes as a more manageable alternative. Lipid-carriers can be engineered for various drugs and polymerized in situ into bilayers [53]; the process decreases the lateral diffusion constant and the permeation coefficient of the membranes, allowing the use of imaging techniques. Further, coupled to graphene-based nanoelectrodes, fast responses and picomolar detection can be easily achieved (e.g., see [54,55]).

Free standing vesicles, ranging from nano- to micro-dimensions, have been also used as membrane models. Easily formed from lipid solutions through a variety of methods (Table 1), their initial scope involved the study of transport functions and mechanisms, permeation properties, adhesion, and fusion kinetics. Liposomes can be classified according to vesicular size and lamellar structure [56]: unilamellar vesicles come as small (20–40 nm), medium (40–80 nm), large (100–1000 nm) or giant (>1000 nm) structures; oligolamellar vesicles are made up of 2–10 bilayers, whereas multilamellar vesicles have several bilayers. Other classification systems are based on the liposome preparation method or the lipid composition. Although the reconstitution of proteins remains somewhat problematic [20], these mobile structures are more suitable for biomedical applications, such as novel in vivo diagnostics and imaging. Towards that end, more customized formulations have been investigated, mainly on the pharmaceutical field.

The simplest approach to construction is the thin film hydration method and all its variants [56]. The method involves solvent evaporation followed by rehydration in aqueous phase, prior to downsizing and homogenization. The size of the liposomes produced is influenced by the charge of the lipids, the properties of the aqueous phase and the nature of the agitation [57]. Although difficult to scale-up, high lipid concentrations can be used to increase encapsulation rates [61]. Hydrophilic compounds and drugs can be passively incorporated within the liposomes at any stage of preparation, and, according to in vitro studies [62], retained for long periods of time with negligible leakage. Still, only a small fraction of the drugs are successfully encapsulated. Post-treatment of the liposome suspension seems to increase drug penetration. For example, freeze-thaw cycles result in the growth of ice crystals between the layers of the liposome and the formation of holes in the bilayer structure; at such platforms, however, significant drug losses have been observed [63].

Alternatively, solvent dispersion can be used [58]. Reverse-phase evaporation yields inverted micelles [57]; when the organic phase is slowly eliminated, the micelles are disrupted to form small and large-sized liposomes. This method achieved probably the higher passive encapsulation rates (ca. 50%). Earlier methods, such as rapid ethanol or ether injection, yield a narrow distribution of small liposomes; the presence of ethanol, however, remains a problem for drug entrapment [62]. The ether treatment is more time and effort consuming but it minimizes the risk of oxidative degradation [56]. Both approaches can be adapted to supercritical fluid processing, by simply replacing the solvent with supercritical carbon dioxide [60], or to microfluidics-based treatment [57].

The encapsulation of compounds sensitive to denaturation (e.g., proteins or DNA) requires different approaches, such as detergent solubilization [59]. The inclusion of water-soluble moieties is a very simple (one-step procedure) but not easily controlled. The proliposome to liposome approach, on the other hand, provides improved accuracies, higher rates, and sterilization capabilities [57,61].

Active liposome loading provides better capabilities for drug delivery. The establishment of a buffer-induced (e.g., citrate) pH gradient between the interior of the liposome and its surrounding was first demonstrated for catecholamines [64]. The citrate method has been, also, successful for doxorubicin, idarubicin, and daunorubicin; drug release rates differ according to the hydrophobicity index of the compounds [65]. Many variants have been proposed to induce pH gradients, such as the ammonium sulfate, the calcium acetate or the ionophore-induced pH gradient methods [62]; regardless, the tradeoff between drug leakage rates and drug release rates should be carefully balanced. Transition metal complexation is a suitable alternative for drug profiles that are not compatible with pH gradients (e.g., ciprofloxacin, alkaloids and hydrophobic drugs) [66], also, allowing the co-encapsulation of different compounds.

Targeting might possibly require liposome surface functionalization. The most common approach involves anchoring, either in situ, where the lipid-ligand conjugate is added with all other lipid components prior to liposome formation, or as post insertion [67]. Alternatively, post-treatment of liposomes with a variety of chemicals, such as amides, thiols or hydrazones, has been also proposed [68]. Still experimental, several problems with these methods should be addressed, such as uncontrolled ligation and ligand-drug interactions.

The use of colloidal cores covered with lipid bilayer (spherically supported bilayer lipid membranes) offers enhanced capabilities in size dispersion, tenability, and physicochemical stability [69]. Relying either on electrostatic attractions between the core and the liposomes [70] or avidin anchoring [71], these platforms are considered more suitable for studying cell-cell interactions. Using nano-cores, these interactions can be even monitored at the molecular level, e.g., by cryoelectron microscopy [72]; further, the self-assembly process per se can be studied in order to reveal critical parameters for the formation of supported bilayer models [69,70].

## 3. Applications, Applicability, and Trends

### 3.1. Biosensors

The development of biosensors based on lipid membranes or liposome platforms is intriguing for a variety of reasons. The possibility of harnessing natural chemoreception on a simple laboratory set-up and transforming it to a highly sensitive detector is by far the most important drive [30]. The bilayer is the natural host for all sorts of proteinaceous moieties, i.e., the functionality of enzymes, antibodies, and receptors and is best preserved within a biomimicking lipidic construct. The immobilization of these simple biomolecules, or even more complex biochemical systems, can follow simple physisorption or more exact and cumbersome chemical binding processes [13].

As the biophysics of the artificial bilayer supports electrochemical interrogation, the formation of the analyte-bioelement complex changes the electrical characteristics of the membrane, readily recorded by electrochemistry. In a broad sense, a biosensor engineer can select and optimize any given lipid bilayer formulation coupled to a successful (rapid, low-noise, readable) transduction system to produce a general biosensor platform. The incorporation of a variety of bioelements yields a respective variety of sensors. In theory, any analyte can be coupled to a bioelement, and any bioelement can be immobilized on a lipid membrane, providing that aqueous chemistry is used.

The issue of operational stability is critical to device development, along with a demonstration of detection in real samples. Table 2 provides a number of lipid membrane-based biosensors that have been recently reported for environmental monitoring and clinical diagnosis [73,74,75,76,77,78,79,80,81,82,83,84,85,86,87,88,89,90,91,92,93,94,95,96,97]. Most platforms involve electrochemical sensing with supported or polymerized bilayers, but optical or more advanced transduction systems have been reported. Sensing using enzymes allows for simple electrochemical systems. The acetycholinesterase biochemistry has been greatly exploited for the detection of pesticides [79,83], while peroxidases have been proposed for dopamine [84] and hydrogen peroxide [90]. Alternatively, peroxidases can be utilized as redox cascades in glucose oxidase platforms [87]. The interactions between urea-urease [95] and uric acid-uricase [96] have been coupled to nanowires enhancing greatly their sensitivity from previously developed Ag/AgCl platforms. Liposomes have been used to monitor in real time the fibrilization of amyloid-beta protein [74]; the system has been slightly modified with cholesterol to provide a micro-cantilever liposome-based assay for the protein with a detection of 75 nM which is suitable for clinical testing [73].

Langmuir-Blodgett films can be used to monitor enzyme activity in reagentless systems using electrochemiluminescence [86]. The authors used choline oxidase to demonstrate the technique. Following antibody-mediated enzyme immobilization at the surface of a luminol-functionalized bilayer, the catalytic generation of hydrogen peroxide triggers a luminescent reaction that can be optically interfaced. Langmuir-based technology has certainly much more to offer as it facilitates the introduction of different lipid groups and conjugates in each monolayer to yield membranes with entirely new properties.

Immunoplatforms exhibit high selectivity allowing for picomolar detection of chemical pollutants [13]. The detection of proteins, however, or protein fragments [82], is usually prohibited by high noise levels; towards that end the use of graphene nanosheets enabled the detection of D-dimer (a fibrin degradation product) at micromolar levels.

The use of receptors expands the range of analytes that can be detected and simplifies detection strategies. For example, the enzyme detection of carbofuran required the use of air-segmented flow to induce inhibition and reactivation of the protein in order to achieve an indirect signal [78]; when authors switched to calixarene receptors, detection was straightforward and detectability decreased manifold.

Receptors are usually large molecules that tend to increase background noise levels and destabilize the membrane. The use of liposomes and optical detection provided a good solution to this problem and a less than picomolar detectability [76,77,80]. Since receptors are stable enough to sustain thermal polymerization, the use of polymerizable membranes incorporated with receptors during polymerization yielded more robust suspended biosensor platforms for toxins [54,55], dopamine [85], ephedrine [85] or naphthalene acetic acid [91].

The exploitation of nano-tools in membrane preparation and signal transduction, also allowed measurements in real samples. Recently, a methaemoglobin biosensor was proposed for measuring nitrites in soil samples [92]. A concanavalin A liposome biosensor could measure and differentiate glycoproteins in serum using electrochemical impedance spectroscopy [88]. Hydrazine pesticides could be detected and differentiated in water samples using DNA-modified bilayer platforms [89].

Besides supporting the bioelement, a prominent feature of the artificial bilayer systems is that the lipid membrane can provide inherent signal amplification [21,22,27,45]. Biosensors are devices that use a biochemical interaction to ‘measure’ the analyte and a transduction system to translate the biochemical signal into an electrical one [30]; an array of signal amplification schemes is usual to most non-membranous platforms. The use of lipid bilayers as an interface between the biochemical interaction and the transduction enables the exploitation of the membrane dynamics: interaction between the bioelement and the analyte changes the biochemical system at the vicinity of the membrane, thus forcing alterations of the dipolar potential and/or the surface charge density and/or transmembrane potential and/or the molecular packing and fluidity, which lead to a significant ion current increase between the two sides of the bilayer [13]. In effect, biochemical changes trigger membrane changes so that the bilayer converts the biochemical signal into an electrochemical one and amplifies it at the same time.

This property of the membrane has been used for detecting analytes without bioelement incorporation. Triazine herbicides exhibit a lipophilicity profile that allows them to adsorb onto lipid membranes creating aggregates that tend to interdigitate with the lipid molecules. These changes alter the molecular packing of lipids producing membrane defects that increase the transmembrane ion current [94]. Similarly, atenolol [74] and vanillin [97] could be measured at micromolar levels [75].

Channel-forming toxins engage a nanopore-formation mechanism to allow the selective and regulated movement of water-soluble moieties and macromolecules. Coupled to lipid platforms, many opportunities arise for sensing [98]. Kasianowicz and Bezrukov [99] demonstrated a relevant strategy based on analyte-induced reversible interruptions of ion current through an α-hemolysin pore in a model membrane. The precision with which these current changes were resolved allowed for a detailed characterization of the analytes or the phenomena studied, including charge, structure, sequence, and kinetics (for more details see [100]). Small peptides, such as gramicidin A of *Bacillus Brevis*, can be chemically modulated as ion switches. Nikolelis et al. [101] utilized gramicidin A channels in metal-supported bilayer lipid membranes to detect ammonium ions; platelet-activating factor was used to modulate the ion pathway and decrease detection limits manifold. Large peptides, such as *a*-hemolysin, aerolysin, lysenin or anthrax toxin, are more suitable for stochastic resistive-pulse single molecule sensing [100]. This technique is crucial for next-generation DNA sequencing or highly sensitive detection of various analytes.

The current trends in nanopore engineering are expected to support the development of more rugged devices for more analytes or even multi-analyte sensors. Yet, a certain disadvantage of lipid membrane biosensors is the lack of suitable mathematical models and simulations [13]. It is true that modeling the complex, ever changing nature of the bilayer, including ion transport phenomena and complex molecular interactions, is quite a challenge [30]. The use of computer simulations may become indispensable in the near future, for optimizing sensor performance and shortening response times, especially when using bi- and tri-enzyme systems or multi-arrays [23,53].

### 3.2. Drug Discovery, Delivery, and Testing

Drugs usually target intracellular reaction sites. Thus, drug-lipid membrane interactions occur at some point, affecting, sometimes severely, drug pharmacokinetics (distribution, accumulation, transport) and efficacy [102]. For example, Michot et al. [103] recorded different intracellular accumulation rates for four structurally similar quinolones (ciprofloxacin, levofloxacin, garenoxacin, and moxifloxacin). When the system was studied with Langmuir-Blodgett model membranes, it was shown that quinolones exert a condensing effect on the bilayer; the strength of the effect differed between the quinolones, as even small differences in their molecular structure alter the lipophilicity of each compound [104].

Azithromycin, on the other hand, interacts selectively only with some types of lipids. Using vesicles, morphometric analysis showed that the drug induces membrane fluctuations in phospholipid but not in sphingomyelin bilayers [105]. Microcinematographic testing further revealed that azithromycin decreases the cohesion between the lipids, thus altering membrane elasticity [106]. A very useful mechanism for liposome-drug delivery was revealed, i.e., changing membrane elasticity in order to facilitate the binding of therapeutic agents.

The estimation of drug efficacy at the early development phases is usually based on partition coefficient values using a two-phase solvent system (e.g., octanol and water); the results, however, may be inconclusive, especially when charged drug molecules are tested [107]. Studies with membrane models, mainly liposomes, instead, have proved to be more relevant and more suitable, as ionic interactions can be readily determined [102]. For example, rifampicin and dibucaine, both ionized at physiological pH, showed similar values when tested with conventional methods but significant differences were recorded in both, anionic and zwitterionic liposomes [108]; the anionic rifampicin does not interact with anionic lipids, whereas the cationic dibucaine does.

Model membranes have been also used to study passive mechanisms of drug transport. Working with amphiphilic drugs, such as haloperidol, Baciu et al. [109] showed that amphiphiles catalyze the hydrolysis of ester links within phospholipid films to yield mono-chain lipids that form micelles and carry the drug across the membrane.

Drug delivery systems currently rely on polymeric coatings, such as chitosan or dextrans. These coatings affect lipid-drug interactions to various degrees and extent. Langmuir-Blodgett models showed, for instance, that chitosan interacts electrostatically with the lipid head groups and may prevent drugs entering the cell [110].

The delivery of reactive plasma species into cancerous cells is still an ongoing project, studied intensively with membrane models almost to an atomic level [111]. A synergistic effect between electric field fluctuations and lipid oxidation induces the formation of pores that carry the reactive species inside the cell.

Nanocolloids are inorganic dispersions that exert size-dependent properties of quantum confinement, supermagnetism, and surface plasmon resonance. The targeting of otherwise inaccessible intracellular sites with these materials, functionalized according to needs, may provide many opportunities for in vivo imaging and testing. The interactions, however, of nanocolloids with biological systems is neither controllable nor straightforward. Nanocolloids yield surfaces with very high energy leading in vitro in uncontrolled aggregation of the particles [112]. That makes them more susceptible to fouling in vivo, i.e., to non-specific binding of proteins. Further, the presence of the colloidal particles in the cell may trigger a number of cellular responses that could disrupt the functionality of the colloidal medium. Surface modifications of the nanocolloids solve these problems but there is no single treatment for all problems. For example, the adsorption of biologically inert proteins (e.g., albumin) or bioactive functionalized dextrans on the surface of the nanocolloids increases the resistance to protein fouling but does not address the problem of aggregation [113]. The use of an outer coating with water-soluble polymers reduces the tendency of the nanocolloids to aggregate but does not prevent non-specific binding [114]. The use of liposomes and lipid microspheres may offer biocompatible and bio-inert surfaces [115], although stability issues remain a consideration [116]; polymerized membranes enhance structural stability without affecting biocompatibility [117]. Notwithstanding, the production of supramolecular assemblies of high complexity, decorated with a variety of functionalities, may support a wide range of applications and provide attoliter detection capabilities [118]. Further, quantum dots encapsulated in copolymer micelles demonstrated in vivo imaging; conjugated to DNA, these assemblies can support lineage-tracing experiments in embryogenesis [119].

Ion channels are attractive targets for drug discovery, yet difficult to screen. The monolayer opposition technique in its low-noise version produced platforms suitable for single channel and single molecule studies. Ion channels can be incorporated into model membranes from a micellar solution or fused with liposomes [120]. After the channel is incorporated into the membrane, the ion current can be induced by applying external voltage or other driving force. Kullman et al. [121] demonstrated single maltoporin channels reconstituted into planar lipid bilayers; using low-pass filters, the researchers could record small ion currents through the channel, induced by single sugar molecules. Low-pass filtering of the single channel signal produces a significant loss of time resolution leaving fast gating events undetectable. White et al. [122] proposed an alternative membrane architecture, consisting of a glass-nanopore supported monolayer and a lipid bilayer suspended across a small orifice; this platform allows for low-noise and higher bandwidth recordings. Kawano et al. [123] used droplet interface bilayers to achieve parallel, single-channel recordings from reconstituted native and mutated ion channels. Recently, the measurement of Förster resonance energy transfer (FRET) in single molecules embedded in lipid nanodiscs enabled the real time monitoring of the structural changes that occur upon channel activation and inhibition [124].

Drug permeability testing remains indispensable for drug development albeit largely debated. Within the various testing approaches available and in use, there exist differences in assay performances and tissues used [125]. Membrane model-based assays are proposed as a suitable approach, provided the methods are standardized. The non-cellular parallel artificial membrane assay uses a freely suspended bilayer in a 96-well microtiter plate to monitor passive permeability [126]. Liposomes have been, also, suggested, dispersed into the pores and the surface of 24-well titre [127]. A demonstration of the suitability of the assay has been also reported through a thorough study [128].

### 3.3. Tools in Research

The use of membrane models in electrophysiology and biochemistry research gained momentum after the recording of a single ion channel was reported [129]. An effort dating back to the mid-1970s, now allows the kinetic behavior of ion channels to be studied using a well-established protocol [130]. Scanning electrochemical microscopy has been recently used to study the dynamic behavior of channel activity in bilayers supported on glassy carbon electrodes [131]. Perchlorate anions were used as stimuli and ruthenium (II) complex cations as probing ions; in the absence of stimuli, the channel was closed. The rate constant could be evaluated by comparing the experimental ion current data with a theoretical model under different stimuli concentrations and externally controlled substrate potentials.

The elucidation of the mechanics and the mechanisms behind peptide-forming voltage-gated channels are largely based on data reported for alamethicin [132] and melittin channels [133]. Under certain experimental conditions and when the peptides occupy one side of the membrane, they both exhibit two relaxation processes: a fast process with weakly voltage-dependent conductance, followed by a slower process with strong voltage-dependent conductance. Both processes have been recently mathematically modelled [134]; the data treatment proposed, relying on nucleation and growth of clusters from monomers, fits satisfactorily the experimental data and also predicts the two conductance regimes.

Protein-lipid interactions and in-membrane functionality has been extensively studied under the scope of developing biosensors. Through the variety of approaches used for protein immobilization and the studies performed in order to provide a justified basis for the mechanism of signal generation, much information has been released, especially as regards conformational changes [135]. Further, recent advances in Langmuir-Blodgett techniques might offer the means to completely simulate physically cell membranes of *E. coli* and mitochondria for studying in vitro complex processes [136].

The elucidation of physiological responses remains an ongoing struggle. For example, the heat shock response (or stress response) was originally attributed to protein denaturation [137]. However, the response might occur in the absence of denatured proteins. A new hypothesis implicated the plasma membrane as a regulator of the response, especially in mild cases, such as fever. Using model membranes [138], a noticeable increase in the sensitivity of transient receptor channels has been recorded at mild heat shock, possibly due to the thermotropic behavior of the lipid bilayer: even small increases in temperature might affect gating events manifold.

The role of sphingosine ceramides in the regulation of skin permeability was recently clarified using lipid membrane models. Školová et al. [139] used model stratum corneum lipid membranes composed of ceramide, lignoceric acid, cholesterol, and cholesteryl sulfate to study the thermotropic and structural behavior of the membrane with respect to the chain length of the ceramides. It was demonstrated that long acyl chain ceramides with C4 hydroxylation increase membrane permeability, whereas ceramides of equal chain length but with C4 unsaturation exert no such effect. Infrared spectroscopy and X-ray diffraction revealed that C4-hydroxylation decreases membrane packing, locally resulting in phase separation of the bilayer. C4-unsaturation, on the other hand, it stabilizes membranes through hydrogen bonding. It was thus concluded that the structural differences of ceramides drive aggregation phenomena and membrane phase shifting to regulate water loss.

### 3.4. Current Trends and Future Perspectives

Neuroscience and neuro-engineering studies with model membranes managed to recreate in vitro and monitor the formation of functional synapses. At an earlier approach, Baksh et al. [140] noticed that bilayer-neuroligin-1 beads activated neuronal cells to form presynaptic nerve terminals at the contact point; replacing the bilayer with polycarbonate beads did not provide any activation although the neuroligin-1 binding activity was preserved. Gopalakrishnan et al. [141] demonstrated presynaptic vesicle accumulation on bilayer lipid membranes supported on silica beads (spherically supported membranes). It was later shown in vitro that the existence of lipid microdomains regulates axonal guidance to yield stable presynaptic contacts when interfaced with neurons [142]; further, it seems that specific functional groups and lateral organizations of the membrane might facilitate synaptic connections. These membrane platforms can interact with living cells and provide a means to investigate the role of membrane heterogeneity in a variety of cellular events. More importantly, the versatility, the tunability, and the biocompatibility of lipid platforms can be adapted to neuro-engineering applications, possibly including artificial synapse formation and synaptogenesis in vivo [141].

Although quite presumptive at the present time, the development of artificial cells to substitute natural ones is lately starting to attract much attention. The integration of non-living components into a cell-like structure that mimics one or more (but certainly only a few) features and functions of the natural cell, is considered more feasible than the creation of a structure that could replace successfully a natural cell [143]. The basic concept of introducing cellular components inside a liposome is old and well-established. The idea of using the liposome as a bioreactor for producing macromolecules out of these cellular components, i.e., for hosting metabolic activity, was demonstrated in 1995: Oberholzer et al. [144] encapsulated polynucleotide phosphorylase and eight different reagents into liposomes to carry out a polymerase chain reaction. A few years later, the same group demonstrated high yield protein biosynthesis within liposomes incorporating the ribosomal complex and all components necessary for protein expression [145]. Yu et al. [146] advanced protein synthesis in liposomes one step further by engineering a complex gene expression network inside giant lipid vesicles; the protein produced was similar as per all aspects to that expressed in natural cells. Kuruma et al. [147] demonstrated the in-liposome production of two proteins and their subsequent function in situ as catalysts for the production of phosphatidic acid. Similarly, Scott et al. [148] demonstrated cell-free phospholipid biosynthesis. Nourian et al. [149] reconstituted a gene expression machinery within a liposome; necessary nutrients and components were supplied in the outside environment and the cell was engineered as an exchange platform to enable the uptake of all necessary components.

**Figure 6:**
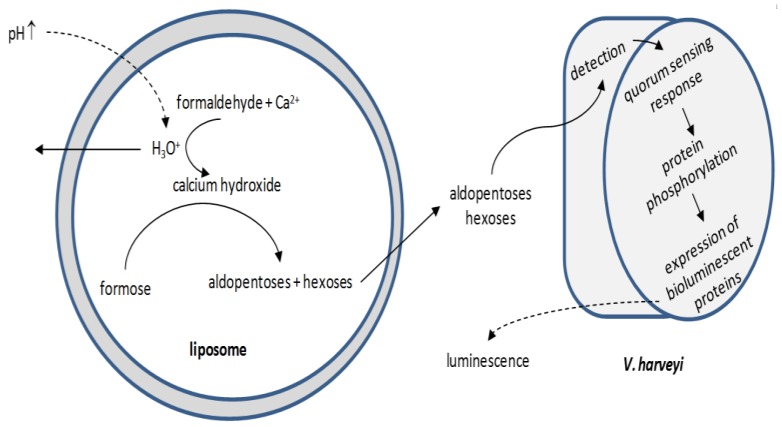
Simplified schematic of liposome-mediated *V. harveyi* bioluminescence induction. The increase in the pH value at the suspension medium triggers the production of sugars inside the vesicles, which cross the membrane and diffuse into the suspension medium. There, they are readily detected by *V. harveyi*; the detection provokes the *Vibrio* cellular mechanism for the production of bioluminescent proteins.

Current efforts are focused in engineering intercellular communication pathways. Gardner et al. [150] developed sugar-producing signaling liposomes: the production of sugars inside the vesicles was triggered by increasing the pH at the suspension medium; the sugars diffused into the medium, detected by *V. harveyi* and the detection induced a quorum-sensing response resulting in the expression of bioluminescent proteins in the bacterium (Figure 6). Lentini et al. [151] produced a hybrid (natural/synthetic) system in order to expand the sensing capabilities of a natural cell: liposomes detect environmental changes that are undetected by *E. coli* and release a chemical signal to *E. coli* to trigger a cellular response, i.e., *E. coli* is equipped with an artificial guard.

Growth and self-replication are functions that can be engineered in vesicles. Early attempts utilized lipid-lipid interactions for incorporating free lipid molecules into preformed liposomes [152]. When the size of the enlarged vesicles reached a critical value for thermodynamic stability, they split into smaller liposomes. Alternatively, micelles could grow into elongated structures by incorporating free lipid molecules [153]. When shear stress becomes dominant, the micelles rupture to produce many vesicles. Many strategies have been reported for triggering cell division by biological-like pathways [143]. Kurihara et al. [154] used supramolecular machinery to link self-replication of RNA/DNA with liposome self-division: the amplified DNA interacted electrostatically with the cationic lipid wall of the host liposome to destabilize it, finally leading to its splitting in a nearly equivolume manner; the amplified DNA distributed between the two smaller vesicles.

Despite this, a multi-responsive artificial cell has not yet been demonstrated. The multifaceted communication of the lipid wall with its exterior would entail the in situ production and accurate placement of several membrane-bound transporters and protein sensors. The in-liposome production of the sensor peptoids is not challenging but the self-decoration of the bilayer with these moieties is; the placement process should be physically-driven because engineering attempts have proven unsuccessful [155].

## 4. Concluding Remarks

Artificial lipid membranes can be constructed by several methods, stabilized by various means, functionalized in a variety of ways, experimented upon intensively, and broadly utilized in sensor development, drug testing, drug discovery or as molecular tools and research probes. Each platform built with a given technique and methodology has certainly its own advantages for serving the respective scope of the research. As the ability to manipulate membranes improves, more multiplexing platforms incorporating complex protein assemblies are being reported. Although production of the majority of these platforms is still restricted within academic laboratories [13,30], no doubt, the advancement of both, science and technology, will facilitate them being engineered into standardized and robust membrane-based instrumentation for commercial use.

The construction of artificial cells is a newly-introduced field of artificial membrane applicability. Technology is not yet capable to fully reproduce biology but a lot of attention has been already concentrated into these structures, opening up unimaginable opportunities for artificial bilayers. The most profound one is the study of evolution pathways for advancing simple structures to current biology complexity by recreating, step by step, cellular life [143,155]. Further, site-specific bioremediation, personalized medicine, high throughput chemical production, phytostimulation and biofertilization, and many more have certainly much to gain from the potential of artificial cells.

## Figures and Tables

**Table 1:** General methods for the preparation of vesicles and the types of vesicles produced.

Method	Description	Types of Liposomes Produced	Ref.
*Physical dispersion*: lipid film hydration by shaking (Bangham method)	Lipids are dissolved in a mixture of solvents in a round bottom flask; solvent evaporation leaves a thin film at the bottom that subsequently is rehydrated with an aqueous buffer. The compounds to be encapsulated can be added either at the solvent mixture or the aqueous buffer.	multilamellar and giant unilamellar vesicles Size reduction (as post-treatment): small unilamellar vesicles (micro-emulsification, bath or probe sonication followed by ultra centrifugation); oligolamellar and/or large unilamellar vesicles (membrane extrusion); small unilamellar vesicles of complex architecture (freeze-thaw sonication)	[56]
*Physical dispersion*: lipid film hydration by non-shaking	Lipids dissolved in organic solvent are freeze dried prior to addition of aqueous buffer. Alternatively, the film is deposited on electrodes and subsequently hydrated in the presence of anelectric field.	multilamellar and giant unilamellar vesicles Size reduction: as above	[56,57]
*Solvent dispersion*: ethanol or ether injection	Lipids in solvent are mixed with the aqueous phase that contains the components to be encapsulated.	small unilamellar vesicles	[58]
*Solvent dispersion*: reverse phase evaporation	A water-in-oil emulsion is formed; the evaporation of the organic phase produces an aqueous suspension of vesicles.	small and large unilamellar vesicles	[57]
*Detergent solubilization*: micelle–vesicle transition	Detergents are used for the solubilization of lipids in micellar systems; the vesicles are released through dilution, gel chromatography, hollow fiber dialysis, membrane filtration, or adsorption to hydrophobic matrix (resins or dextrins).	multilamellar, oligolamellar, large unilamellar vesicles (dialysis); small unilamellar vesicles (gel chromatography, filtration, adsorption)	[59]
*Proliposomes*: hydration	Proliposomes are formed by drying a lipid solution; solvent removal proceeding with rotary vacuum evaporation, fluidized bed adsorption or spray drying. When diluted in aqueous phase (along with the components to be encapsulated), a vesicle dispersion is produced; encapsulation efficiencies are high and the products can be sterilized.	multilamellar vesicles	[58]
*Supercritical fluid technology*: anti-solvent method and reverse phase evaporation	In the anti-solvent method, the lipids dissolve in supercritical CO_2_ and then precipitate in the form of ultra-fine particles. In reverse phase evaporation, supercritical CO_2_ is used instead of conventional solvents.	multilamellar and giant unilamellar vesicles (anti-solvent method); small and large unilamellar vesicles (reverse phase evaporation)	[60]
*Microfluidic methods*: hydrodynamic focusing, droplets, pulsed jet flow, thin film hydration	Microfluidics offer micro-to nanoliter volumes of vesicles dispersions and precise control over production.	small unilamellar vesicles (micro hydrodynamic focusing); giant unilamellar vesicles (microfluidic droplets and pulsed jet flow microfluidics); large unilamellar vesicles (thin film hydration in microtubes)	[57]

**Table 2:** Bilayer lipid membrane biosensors for environmental monitoring and clinical diagnostics.

Analyte	Biological System/Membrane	Transducer Type	Detection Limit	Ref.
Amyloid-β protein	Cholesterol incorporated liposomes	Micro-cantilever with NiCr thin film strain gauge	75 nM	[73]
Amyloid-β protein, real time continuous monitoring of fibrilization	Liposomes	Micro-cantilever with NiCr thin film strain gauge	1 μΜ	[74]
Atenolol	Polymerized membranes	Ag/AgCl electrodes	20 μM	[75]
Botulinum neurotoxin	Trisialoganglioside functionalized liposomes	Fluorescence	–	[76]
Carbofuran pesticidein foods	Resorcinarene receptor/glass filter supported membranes	Fluorescence	1 nM	[77]
Carbofuran pesticide in foods	Calixarene receptor/polymerized membranes	Graphene-nanosheets- based electrodes	1 μΜ	[78]
Carbofuran pesticide in foods	Acetylcholinesterase/polymerized membranes	Ag/AgCl electrodes	1 nM	[79]
Cholera toxin	Ganglioside GM1/liposomes	Chemiluminescence	0.8 pM	[80]
Cholera toxin in water	Ganglioside GM1/polymerized membranes	Graphene-nanosheets- based electrodes	1 nM	[55]
Cholesterol	Cholesterol oxidase/polymerized membranes	Graphene-nanosheets-based electrodes	0.1 μM	[81]
d-dimer	Antibody/polymerized membranes	Graphene-nanosheets- based electrodes	1 μM	[82]
Dichlorvos pesticide	Acetylcholinesterase/liposome-chitosan nanocomposite	Ag/AgCl electrodes	0.25 μM	[83]
Dopamine	Peroxidase/dithiotreitol supported membranes	Au electrode	2 μM	[84]
Dopamine in human urine	Pirogallolarene receptor/polymerized membranes	Fluorescence	1 nM	[85]
Enzyme activity, reagentless monitoring of	Langmuir-Blodgett membranes	Electro-chemiluminescence	–	[86]
Ephedrine in human urine	Permethoxy receptor/polymerized membranes	Fluorescence	1 nM	[85]
Glucose	Glucose oxidase/microperoxidase functionalized liposomes	Indium-tin oxide (ITO) electrode	8.6 μM	[87]
Glycoproteins in serum	Concanavalin A/liposomes	Electrochemical impedance spectroscopy	not reported	[88]
Hydrazine pesticides in water	DNA/glass filter supported membranes	Ag/AgCl electrodes	78 pM	[89]
Hydrogen peroxide	Peroxidase/polymerized membrane	Electrochemical impedance spectroscopy	0.1 μΜ	[90]
Naphthalene acetic acid in foods	Auxin-binding protein receptor/polymerized membranes	Graphene-nanosheets-based electrodes	10 nM	[91]
Nitrites in soil	Methaemoglobin/metal- supported membranes	Ag/AgCl electrodes	0.9 μg/L	[92]
Polychlorinated biphenyls (arochlor)	Antibody/polymerized membranes	Ag/AgCl electrodes	10 pM	[93]
Saxitoxin in foods and water	Anti-saxitoxin receptor/polymerized membranes	Graphene-nanosheets-based electrodes	1 nM	[54]
Triazine herbicides in water	Metal supported membranes	Ag/AgCl electrodes	15 nM	[94]
Urea	Urease/polymerized membranes	Graphene-nanosheets-based electrodes	1 μM	[95]
Uric acid	Uricase/polymerized membranes	ZnO nanowires-based electrodes	1 μM	[96]
Vanillin in alcoholic beverages and wine	Polymerized membranes	Ag/AgCl electrodes	0.3 μM	[97]
